# C-reactive protein predicts respiratory failure in chronic obstructive pulmonary disease: a cohort analysis from the UK Biobank

**DOI:** 10.7189/jogh.16.04061

**Published:** 2026-02-27

**Authors:** Boyan Zhang, Zhongshang Dai, Qi Jiang, Rui Zhao, Yan Chen

**Affiliations:** 1 The Second Xiangya Hospital of Central South University, Department of Respiratory and Critical Care Medicine, Changsha, China; 2 The Second Xiangya Hospital of Central South University, Department of Infectious Diseases, Changsha, China; 3 Central South University, Research Unit of Respiratory Disease, Changsha, China; 4 Clinical Medical Research Centre for Pulmonary and Critical Care Medicine in Hunan Province, Changsha, China; 5 Central South University, Diagnosis and Treatment Centre of Respiratory Disease, Changsha, China

## Abstract

**Background:**

Respiratory failure (RF) is the leading cause of death in chronic obstructive pulmonary disease (COPD), yet reliable biomarkers for early risk stratification remain unclear. Circulating C-reactive protein (CRP) reflects systemic inflammation, but its prognostic value for incident RF in COPD is controversial.

**Methods:**

A total of 38 933 patients from the UK Biobank with the ratio of Forced Expiratory Volume in 1 second to Forced Vital Capacity (FEV_1_/FVC) < 0.70 but without RF at baseline were included, and a maximum of 17.87 years of follow-up was conducted. Participants were divided into five subgroups based on serum CRP concentration. Kaplan-Meier survival analysis was utilised to assess the correlation between CRP stratification, incident RF, all-cause mortality, and COPD-induced mortality. The dose-response relationship between CRP concentration and incident RF was investigated using Cox proportional hazards regression.

**Results:**

Kaplan-Meier curves showed statistically significant differences in RF across all subgroups throughout the entire follow-up period. Additionally, significant differences were observed between groups concerning all-cause mortality and COPD-induced mortality as well. The Cox proportional hazards model demonstrated a clear dose-response relationship between CRP concentration and RF, even after adjustment for several clinical covariates and systemic inflammation index.

**Conclusions:**

Serum CRP concentration may forecast a high risk of incident RF in patients with COPD, indicating further research on the threshold.

Chronic obstructive pulmonary disease (COPD) is one of the most common chronic respiratory diseases characterised by heterogeneity. Patients typically present with symptoms, such as cough, sputum production, or decreased exercise tolerance [1]. According to the WHO, the number of patients with COPD worldwide has exceeded 380 million [2], with Chinese patients accounting for roughly 25% [3]. Epidemiological studies have shown that COPD has become the third leading cause of death [4]. Consequently, COPD imposes a substantial burden on public health and socio-economic systems.

Respiratory failure (RF), a hallmark of severe acute exacerbation in COPD [1], is also the primary cause of poor prognosis. Studies have shown that RF accounts for nearly 38.3% of deaths in patients with COPD [5]. Simultaneously, RF may contribute to the occurrence and exacerbation of other comorbidities. Existing literature has shown that RF and hypercapnia are risk factors for an unfavourable prognosis of COPD [6]. Given its significant contribution to COPD mortality, effective control of RF is a crucial measure for improving patient outcomes.

Among the multiple underlying causes of RF, inflammation is the most common in patients with COPD [7]. Chronic inflammation can cause airway congestion and collapse, which together gradually lead to irreversible airflow limitation. Meanwhile, acute inflammation is closely associated with acute exacerbations of COPD [8].

C-reactive protein (CRP) is a typical biomarker reflecting systemic inflammation level. Elevation of CRP level in patients with COPD has already been proven to be associated with the frequency of hospitalisation and the occurrence of death events [9]. Available studies have shown that CRP concentration is significantly increased in patients with acute exacerbation and gradually decreases with disease remission [10]. It has also been found that even in the stable phase of COPD, CRP concentration is significantly higher than that in the normal population [11]. Studies above suggest that CRP plays a critical role in the development of patients with COPD.

Although CRP is widely recognised as an inflammatory biomarker in cardiovascular diseases, its association with RF in patients with COPD remains unclear. Some researchers suggest that CRP levels are not elevated in patients with stable COPD [12–14], and the relationship between CRP and all-cause mortality in patients with COPD is also controversial [9,12,15,16]. In addition, the optimal CRP cutoff for predicting prognosis in patients with COPD remains unclear [9]. To date, few studies with long-term follow-up have examined the feasibility and clinical utility of CRP for predicting incident RF in patients with COPD.

Undoubtedly, there is a pressing need to clarify its association with RF, given the unclear and controversial results. Therefore, a maximum 17.87-year retrospective analysis of the prospective cohort study was conducted to assess whether baseline CRP concentration predicts the risk of RF in patients with COPD. Moreover, we included the systemic inflammation index (SII) [17] to provide a more comprehensive assessment of systemic inflammation based on the pathophysiological characteristics of COPD. By analysing these associations, we aim to determine the relationship between baseline CRP concentration and the risk of incident RF in patients with COPD, thereby facilitating early identification of high-risk patients and ultimately trying to improve clinical outcomes.

This study was based on a publicly available large-scale population database, and we fully consent to and support the policy of JoGH’s Guidelines for Reporting Analyses of Big Data Repositories Open to Public [18]. All adherence data are presented in Table S1 in the **Online Supplementary Document**.

## METHODS

### Study design and population

#### Study population

This long-term, retrospective analysis of the prospective cohort study used data from the UK Biobank, which contains more than 500 000 participants and their biospecimens in the UK during 2006–2010. Full study sampling methods for the UK Biobank are described elsewhere [19].

#### Inclusion/exclusion criteria

Participants aged between 40–70 years, whose baseline lung function test reached the criteria of COPD (FEV_1_/FVC < 0.70) but excluded RF (inpatient diagnosis 10th revision of the International Classification of Diseases (ICD-10) codes including J96.0/J96.1/J96.9), were enrolled in this study. The exclusion criteria included: participants with an earlier-than-baseline or baseline diagnosis of RF; participants with other terminal-stage diseases (end-stage renal disease and lung cancer); participants with a calculated survival time of 0 or less; and participants with missing or contradictory data. In conclusion, a total of 38 933 eligible participants were ultimately enrolled in this study.

We also conducted a cohort-based sensitivity analysis, using inpatient ICD-10 codes (J44.0/J44.1/J44.8/J44.9) as the criteria for COPD, and included 12 272 participants in total, applying the same inclusion and exclusion criteria. A detailed screening process is shown in the flowchart in the supplemental material (Figure S1 in the **Online Supplementary Document**).

#### Study goal

This study was conducted to discover the relationship between serum CRP concentration and RF in patients with COPD.

### Data collection

#### Exposure factors and covariates

C-reactive protein concentration (mg/L) was selected as the primary exposure factor derived from the baseline assessment of the UK Biobank. Meanwhile, SII was included as an auxiliary exposure variable for analysis. Multiple clinical covariates, including gender, age, body mass index (BMI), smoking status, pack-years of smoking (smoking index), and FEV_1_/FVC, were included in our analysis to better characterise the population and mitigate substantial bias across study subgroups.

#### Outcomes

Our primary outcome was time to first event, defined as the time from the baseline assessment of lung function to the first RF inpatient diagnosis or administrative censor date set at 1 March 2025, whichever occurred first. In the primary Cox models for incident RF, death before incident RF was treated as a competing risk and censored. Furthermore, to better elucidate the association between CRP and RF and to clarify their implications in patients with COPD, we also examined all-cause and COPD-induced mortality as secondary outcomes, defined by ICD-10 codes for the underlying (primary) cause of death in the database.

### Statistical analysis

#### Grouping and baseline processing

C-reactive protein concentration of the participants was divided into four subgroups (Q_1_–Q_4_) according to the quartile of CRP concentration under 20 mg/L. Participants with CRP over 20 mg/L were grouped separately as Q_5_. Systemic inflammation index was calculated by ‘platelet × neutrophil count/lymphocyte count’ and was divided into four subgroups (Q_1_–Q_4_) by quartiles.

We censored all the participants with missing values on demographic information, CRP and other blood test results, diagnostic information, and lung function results. Those with missing smoking-related indicators were treated as described in the Supplemental Methods and Table S2 in the **Online Supplementary Document**.

#### Descriptive statistics

Baseline characteristics stratified by CRP groups are summarised in **Table 1**, while the risk profile of RF-binary classification groups is presented in **Table 2**. ANOVA and Kruskal-Wallis tests were used to compare the differences between groups in **Table 1**.

**Table 1:** Baseline characteristics stratified on CRP quintiles*

Variables	CRP Q_1_ (n = 9699)	CRP Q_2_ (n = 9577)	CRP Q_3_ (n = 9503)	CRP Q_4_ (n = 9576)	CRP Q_5_ (n = 578)	Overall (n = 38 933)
Sex, n (%)						
*Female*	4554 (47.0)	4240 (44.3)	4306 (45.3)	4647 (48.5)	235 (40.7)	17 982 (46.2)
*Male*	5145 (53.0)	5337 (55.7)	5197 (54.7)	4929 (51.5)	343 (59.3)	20 951 (53.8)
Smoking, n (%)						
*No*	5311 (54.8)	4490 (46.9)	3836 (40.4)	3162 (33.0)	184 (31.8)	16 983 (43.6)
*Yes*	4388 (45.2)	5087 (53.1)	5667 (59.6)	6414 (67.0)	394 (68.2)	21 950 (56.4)
RF, n (%)						
*No*	9623 (99.2)	9491 (99.1)	9348 (98.4)	9337 (97.5)	554 (95.8)	38 353 (98.5)
*Yes*	76 (0.8)	86 (0.9)	155 (1.6)	239 (2.5)	24 (4.2)	580 (1.5)
Death, n (%)						
*No*	8663 (89.3)	8284 (86.5)	7951 (83.7)	7405 (77.3)	386 (66.8)	32 689 (84.0)
*Yes*	1036 (10.7)	1293 (13.5)	1552 (16.3)	2171 (22.7)	192 (33.2)	6244 (16.0)
COPD-induced death, n (%)						
*No*	9651 (99.5)	9503 (99.2)	9399 (98.9)	9406 (98.2)	560 (96.9)	38 519 (98.9)
*Yes*	48 (0.5)	74 (0.8)	104 (1.1)	170 (1.8)	18 (3.1)	414 (1.1)
CRP	0.45 (0.08–0.73)	1.06 (0.74–1.49)	2.09 (1.50–3.02)	5.02 (3.03–19.99)	27.22 (20.00–78.90)	1.51 (0.08–78.90)
Age	60.00 (40.00–70.00)	61.00 (40.00–70.00)	61.00 (40.00–70.00)	62.00 (40.00–70.00)	62.00 (40.00–70.00)	61.00 (40.00–70.00)
BMI	24.26 (12.74–53.78)	25.93 (15.34–49.32)	27.03 (16.44–58.26)	28.29 (14.59–57.21)	27.20 (13.34–50.65)	26.23 (12.74–58.26)
Smoking index	0.00 (0.00–182.00)	4.50 (0.00–301.00)	12.00 (0.00–265.00)	19.25 (0.00–215.00)	21.00 (0.00–168.00)	8.25 (0.00–301.00)
FEV_1_/FVC	0.66 (0.15–0.70)	0.66 (0.13–0.70)	0.66 (0.07–0.70)	0.65 (0.03–0.70)	0.65 (0.13–0.70)	0.66 (0.03–0.70)
Albumin	45.45 (30.72–57.59)	45.09 (30.57–56.43)	44.82 (26.00–58.34)	44.21 (23.42–56.42)	42.93 (34.80–50.81)	44.87 (23.42–58.34)
Haematocrit	41.20 (20.11–60.40)	41.80 (20.40–57.91)	41.80 (0.05–57.30)	41.51 (16.68–71.09)	40.58 (20.85–52.40)	41.54 (0.05–71.09)
Haemoglobin	14.20 (6.15–19.38)	14.41 (7.00–19.31)	14.40 (0.11–20.30)	14.30 (6.00–20.08)	13.95 (9.92–17.20)	14.33 (0.11–20.30)
Platelet	237.90 (27.00–963.20)	244.20 (8.20–773.70)	249.70 (0.90–824.00)	259.00 (4.80–843.00)	262.00 (48.80–554.10)	247.50 (0.90–963.20)
WBC	6.30 (1.45–102.30)	6.80 (2.26–93.70)	7.10 (0.21–38.10)	7.73 (1.05–73.65)	8.49 (3.03–19.24)	6.96 (0.21–102.30)
Neu†	3.76 (0.04–13.25)	4.10 (0.04–14.20)	4.35 (0.07–17.08)	4.86 (0.00–23.90)	5.70 (0.70–12.58)	4.26 (0.00–23.90)
Neu‡ (%)	60.50 (2.43–92.90)	61.20 (0.78–95.70)	61.80 (1.43–94.00)	63.40 (0.00–94.00)	67.65 (16.80–92.70)	61.80 (0.00–95.70)
Eos†	0.13 (0.00–3.53)	0.15 (0.00–2.97)	0.17 (0.00–2.61)	0.19 (0.00–9.60)	0.19 (0.00–2.45)	0.16 (0.00–9.60)
Eos‡ (%)	2.18 (0.00–39.90)	2.27 (0.00–25.74)	2.30 (0.00–33.96)	2.25 (0.00–100.00)	2.01 (0.00–18.10)	2.25 (0.00–100.00)
Baso†	0.02 (0.00–1.10)	0.02 (0.00–2.00)	0.02 (0.00–1.40)	0.03 (0.00–2.10)	0.03 (0.00–0.60)	0.02 (0.00–2.10)
Baso‡ (%)	0.44 (0.00–12.40)	0.42 (0.00–33.80)	0.43 (0.00–16.40)	0.43 (0.00–16.30)	0.40 (0.00–8.40)	0.43 (0.00–33.80)
Lym†	1.80 (0.11–92.30)	1.90 (0.28–82.90)	1.93 (0.03–33.20)	2.00 (0.00–60.63)	1.80 (0.54–10.43)	1.90 (0.00–92.30)
Lym‡ (%)	28.89 (5.19–90.20)	28.21 (2.87–89.00)	27.70 (3.50–87.30)	26.30 (0.00–89.90)	21.44 (5.69–63.70)	27.70 (0.00–90.20)
Mono†	0.42 (0.00–4.67)	0.48 (0.00–7.90)	0.50 (0.00–10.37)	0.50 (0.00–12.60)	0.60 (0.05–1.66)	0.49 (0.00–12.60)
Mono‡ (%)	6.98 (0.26–64.80)	6.96 (0.26–74.30)	6.90 (0.06–90.50)	6.73 (0.00–87.00)	7.11 (0.60–26.90)	6.90 (0.00–90.50)
SII	495.00 (5.19–5125.09)	526.28 (6.76–7968.00)	555.22 (5.10–12 540.00)	618.79 (7.78–7878.66)	832.18 (75.32–5368.00)	548.22 (5.10–12 540.00)
Survival time	16.02 (0.04–17.86)	15.97 (0.04–17.86)	15.95 (0.06–17.87)	15.79 (0.01–17.87)	15.33 (0.08–17.74)	15.94 (0.01–17.87)

Baso – basophil, BMI – body mass index, COPD – chronic obstructive pulmonary disease, CRP – c-reactive protein, Eos – eosinophil, FEV_1_ – forced expiratory volume in 1 s, FVC – forced vital capacity, Lym – lymphocyte, Mono – monocyte, Neu – neutrophil, Q – quartile, RF – respiratory failure, SII – systematic inflammation index, WBC – white blood cell

*Values are presented as median (range) unless specified otherwised.

†Count of haemocytes.

‡Percentage of haemocytes.

**Table 2:** Baseline characteristics stratified on incident RF*

Variable	No RF (n = 38 353)	RF (n = 580)	Overall (n = 38 933)
Sex, n (%)			
*Female*	17 749 (46.3)	233 (40.2)	17 982 (46.2)
*Male*	20 604 (53.7)	347 (59.8)	20 951 (53.8)
Smoking, n (%)			
*No*	16 906 (44.1)	77 (13.3)	16 983 (43.6)
*Yes*	21 447 (55.9)	503 (86.7)	21 950 (56.4)
Death, n (%)			
*No*	32 516 (84.8)	173 (29.8)	32 689 (84.0)
*Yes*	5837 (15.2)	407 (70.2)	6244 (16.0)
COPD-induced death, n (%)			
*No*	38 043 (99.2)	476 (82.1)	38 519 (98.9)
*Yes*	310 (0.8)	104 (17.9)	414 (1.1)
CRP group, n (%)			
*Q_1_*	9623 (25.1)	76 (13.1)	9699 (24.9)
*Q_2_*	9491 (24.7)	86 (14.8)	9577 (24.6)
*Q_3_*	9382 (24.5)	156 (26.9)	9538 (24.5)
*Q_4_*	9303 (24.3)	238 (41.0)	9541 (24.5)
*Q_5_*	554 (1.4)	24 (4.1)	578 (1.5)
CRP	1.50 (0.08–78.90)	2.63 (0.15–49.97)	1.51 (0.08–78.90)
Age	61.00 (40.00–70.00)	63.00 (40.00–70.00)	61.00 (40.00–70.00)
BMI	26.22 (12.74–58.26)	27.25 (12.81–50.80)	26.23 (12.74–58.26)
Smoking index	7.75 (0.00–301.00)	35.00 (0.00–265.00)	8.25 (0.00–301.00)
FEV_1_/FVC	0.66 (0.03–0.70)	0.61 (0.27–0.70)	0.66 (0.03–0.70)
Albumin	44.88 (23.42–58.34)	44.19 (36.51–55.54)	44.87 (23.42–58.34)
Haematocrit	41.53 (0.05–71.09)	42.08 (27.20–57.05)	41.54 (0.05–71.09)
Haemoglobin	14.33 (0.11–20.30)	14.50 (9.20–19.38)	14.33 (0.11–20.30)
Platelet	247.35 (0.90–963.20)	254.50 (64.90–640.00)	247.50 (0.90–963.20)
WB	6.94 (0.21–102.30)	7.94 (3.24–25.43)	6.96 (0.21–102.30)
Neu†	4.24 (0.04–17.08)	5.05 (0.07–23.90)	4.26 (0.04–23.90)
Neu‡	61.80 (0.78–95.70)	63.45 (0.93–94.00)	61.80 (0.78–95.70)
Eos†	0.16 (0.00–3.53)	0.18 (0.00–3.25)	0.16 (0.00–3.53)
Eos‡	2.25 (0.00–39.90)	2.20 (0.00–25.86)	2.25 (0.00–39.90)
Baso†	0.02 (0.00–2.10)	0.03 (0.00–0.79)	0.02 (0.00–2.10)
Baso‡	0.43 (0.00–33.80)	0.44 (0.00–10.72)	0.43 (0.00–33.80)
Lym†	1.90 (0.03–92.30)	2.02 (0.20–8.55)	1.90 (0.03–92.30)
Lym‡	27.73 (2.87–90.20)	25.50 (3.02–89.00)	27.70 (2.87–90.20)
Mono†	0.49 (0.00–12.60)	0.56 (0.06–7.88)	0.49 (0.00–12.60)
Mono‡	6.90 (0.06–90.50)	7.02 (0.50–76.70)	6.90 (0.06–90.50)
SII	547.39 (5.10–12 540.00)	611.02 (6.76–7878.66)	548.22 (5.10–12 540.00)

Baso – basophil, BMI – body mass index, COPD – chronic obstructive pulmonary disease, CRP – c-reactive protein, Eos – eosinophil, FEV_1_ – forced expiratory volume in 1 s, FVC – forced vital capacity, Lym – lymphocyte, Mono – monocyte, Neu – neutrophil, Q – quartile, RF – respiratory failure, SII – systematic inflammation index, WBC – white blood cell

*Values are presented as MD (range) unless specified otherwise.

†Count of haemocytes.

‡Percentage of haemocytes.

#### Survival analysis

We censored the data from the administrative end date onward. Subsequently, we conducted a survival analysis based on CRP subgroups. Kaplan-Meier curves and log-rank test were constructed to examine time-to-event differences among CRP groups and RF, as well as all-cause and COPD-induced mortality. Log-rank test with Bonferroni correction was carried out to compare pairwise among CRP groups. ‘*P* < 0.0001’ is displayed if the corrected *P*-value is less than 1 × 10^−4^.

#### Cox regression analysis

Univariate Cox regression analyses were performed for CRP and SII separately to estimate the hazard ratio (HR), 95% confidence interval (CI), and Wald test *P*-value for each non-reference level relative to the reference group (Q_1_). Forest plots were constructed to better illustrate the level of associated risk. Furthermore, we constructed a multivariate Cox regression model with CRP, adjusted for clinical covariates and SII, to evaluate the independent effect of CRP on RF. The proportional hazard (PH) test and Schoenfeld's residual error plot were used to assess whether a time-dependent trend in CRP exists.

#### Fine-Grey models

We used a Fine-Grey model to examine competing risks of death as a supplementary method to strengthen the results of the Cox regression models, and the sub-distribution hazard ratio (SHR) of CRP-to-RF was calculated and presented in the supplemental material (Table S4 in the **Online Supplementary Document**).

#### Restricted cubic splines

A restricted cubic spline (RCS) was used as a supplementary verification method to investigate the association between CRP concentration and RF when treating CRP as a continuous variable. We solely utilise RCS as a secondary analytical method because our main focus should be on the CRP-RF relationship itself, rather than on the specific cut-off point, as it is the key outcome of our research.

All analyses were conducted with *R* statistical software, v.4.5.1 (R Foundation for Statistical Computing, Vienna, Austria).

## RESULTS

### Baseline characteristics

**Figure 1:**
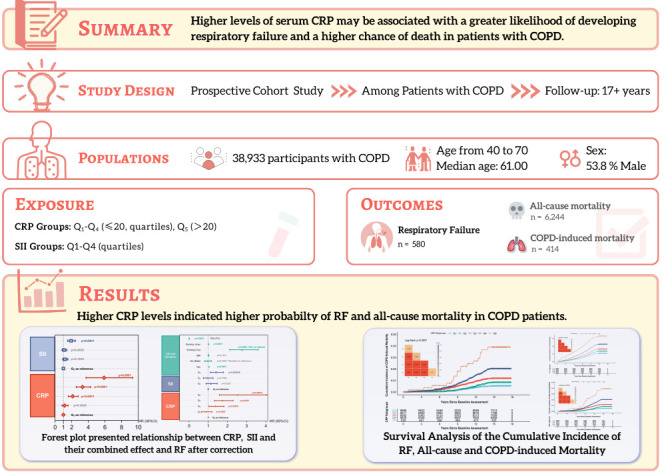
Graphical illustration.

A total of 38 933 participants, with a mean age of 61.00 years, were included in this study and followed for up to 17.87 years (**Figure 1**). All participants were divided into five groups according to CRP levels. Our results demonstrate a significant difference in incident RF among the five CRP groups (*P* < 0.0001). Additional relevant baseline data are presented in **Table 1**.

### RF incidents

During the whole follow-up period, 580 RF cases were observed, including 556 cases with CRP<20 mg/L (cumulative incidence rate = 1.45%) and 24 cases with CRP over 20 mg/L (cumulative incidence rate = 4.15%). The ratio of cumulative incidences between Q_5_ and Q_1–4_ is 2.862. The median CRP was 1.50 mg/L (0.08–78.90 mg/L) in the group without RF and 2.63 mg/L (0.15–49.97 mg/L) in the group with RF. Additional baseline information for the RF-binary groups is presented in **Table 2**.

### Association between CRP level and incident RF

#### Kaplan-Meier survival analysis

**Figure 2:**
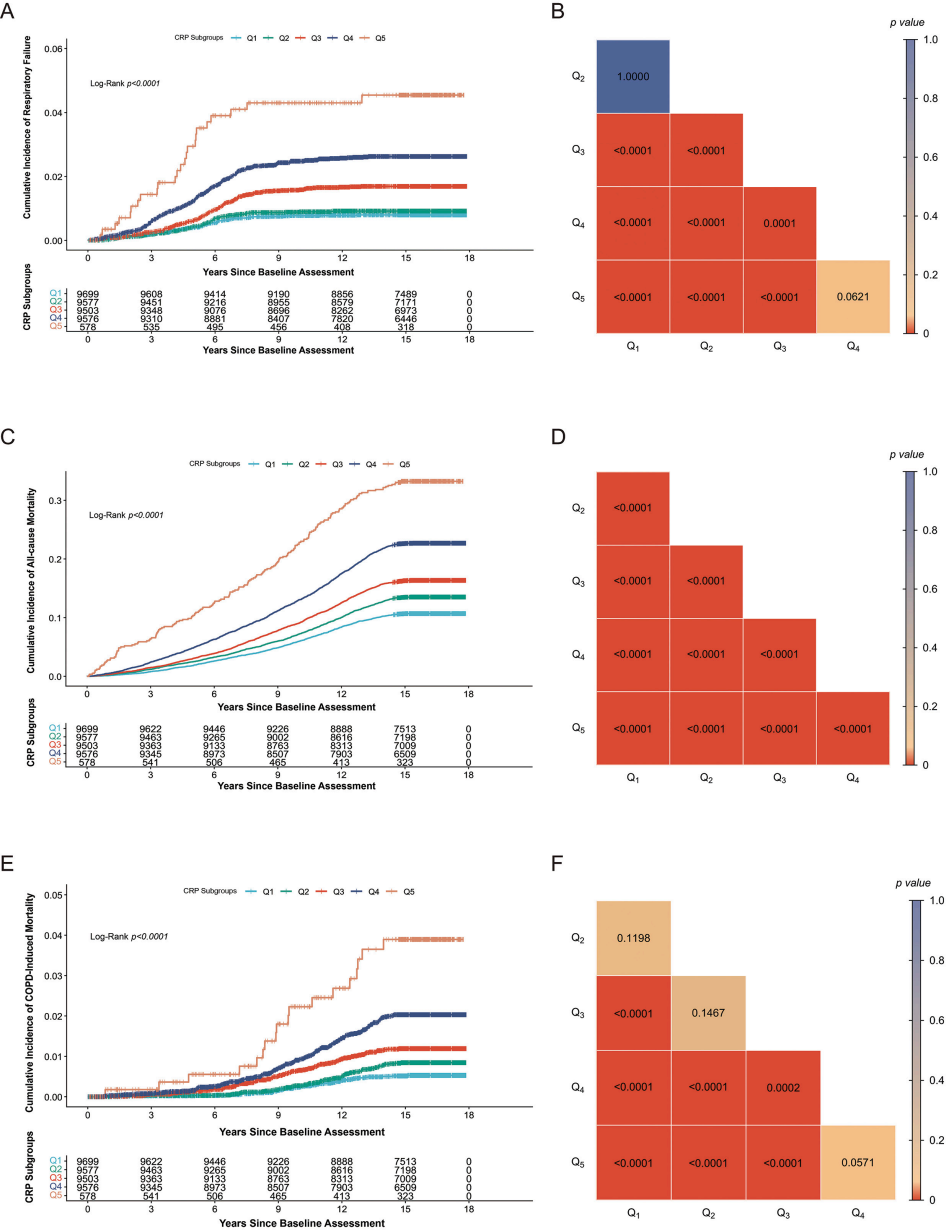
Survival analysis between CRP groups and outcomes. **Panel A**. Kaplan-Meier curve between CRP and incident RF. **Panel B**. Pairwise comparison ended with incident RF. **Panel C**. Kaplan-Meier curve between CRP and all-cause mortality. **Panel D**. Pairwise comparison ended with all-cause mortality. **Panel E**. Kaplan-Meier curve between CRP and COPD-induced mortality. **Panel F**. Pairwise comparison ended with COPD-induced mortality. COPD – chronic obstructive pulmonary disease, CRP – C-reactive protein, RF – respiratory failure.

During the entire follow-up period, the Kaplan-Meier survival curves for each CRP group did not exhibit significant crossing, and the probability of RF increased over time across all groups with a statistically significant difference (χ^2^ = 162.72, Log*-*Rank *P* < 0.0001) (**Figure 2**, Panel A). The increase was steepest in Q_5_, followed by Q_4_ and Q_3_, and slowest in Q_2_ and Q_1_, indicating a clear dose-response relationship between the risk of RF and CRP concentration.

At 15 years of follow-up, the cumulative incidence of RF increased gradually from 10.7% (95% CI = 10.1–11.3%) in Q_1_ to 33.43% (95% CI = 29.46–37.18%) in Q_5_. After Bonferroni correction, pairwise comparisons revealed a significant difference between most subgroups, except for Q_1_ & Q_2_, and Q_4_ & Q_5_ (*P* > 0.05) **(****Figure 2**, Panel B).

To further elucidate the results, given the well-documented clinical observations [20], we included all-cause mortality as a secondary outcome and examined its association with CRP levels. Kaplan-Meier curves for all-cause mortality showed a time-dependent increase in each group, with a statistically significant difference across groups (χ^2^ = 729.78, Log-Rank *P* < 0.0001) (**Figure 2**, Panel C). These observations suggest a similar dose-response relationship, which was more pronounced in the group with higher CRP concentrations. After Bonferroni correction, significant differences were observed across all subgroups (*P* < 0.05) (**Figure 2**, Panel D).

Given the significance of CRP in all-cause mortality analysis, we then introduce COPD-induced mortality to further evaluate the effect of CRP in the context of COPD. For COPD-induced mortality, the Kaplan-Meier curve also shows a similar upward trend with CRP levels (χ^2^ = 126.34, Log-Rank *P* < 0.0001) (**Figure 2**, Panel E), and pairwise comparisons after Bonferroni correction indicated statistically significant differences except for Q_1_ & Q_2_, Q_2_ & Q_3_, and Q_4_ & Q_5_ (*P* > 0.05) (**Figure 2**, Panel F).

#### Cox regression analysis

**Figure 3:**
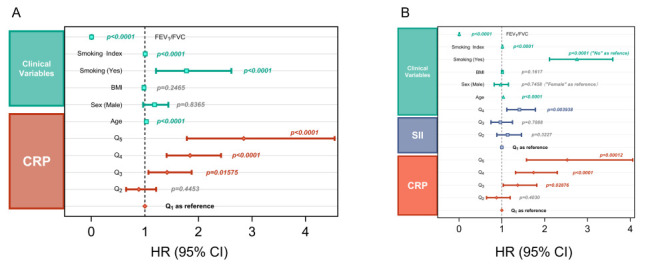
Cox proportional risk regression forest plot. **Panel A**. Univariate Cox regression analysis showed a dose-response relationship between CRP and incident RF. **Panel B**. Multivariate Cox regression analysis after the correction of clinical variables and treating SII as a covariate, indicating the risk status of CRP after correlation of SII and clinical variables. CI – confidence interval, CRP – C-reactive protein, HR – hazard ratio, RF – respiratory failure, SII – systemic inflammation index.

We performed univariate Cox regression analysis to further quantify the association between CRP and incident RF (**Figure 3**, Panel A, **Table 3**, Panel A). In parallel, SII was introduced to better characterise its relationship with systemic inflammation. We observed that higher CRP concentrations were associated with an increased risk of incident RF. Compared with the lowest CRP group (Q_1_), the HRs of RF in the Q_3_, Q_4_, and Q_5_ groups increased to 2.11 (95% CI = 1.60–2.78, *P* < 0.001*)*, 3.31 (95% CI = 2.56–4.28, *P* < 0.001), and 5.90 (95% CI = 3.73–9.34, *P* < 0.001), however, in Q_2_ subgroup we observed no significant difference (HR = 1.15; 95% CI = 0.848–1.57, *P* = 0.3628). For SII, we observed a statistically significant difference only in the highest subgroup (HR = 1.93; 95% CI = 1.54–2.43) compared with the SII Q_1_.

**Table 3:** Baseline characteristics stratified on incident RF

Variables	Models*	Groups	HR	SD	CI-low	CI-high	*P*-value
CRP†	A	Q_2_	1.154	0.1574	0.8476	1.571	0.3628
		Q_3_	2.113	0.1400	1.606	2.780	<0.0001
		Q_4_	3.309	0.1317	2.556	4.283	<0.0001
		Q_5_	5.900	0.2342	3.729	9.336	<0.0001
SII†	A	Q_2_	1.195	0.1280	0.9300	1.536	0.1635
		Q_3_	1.100	0.1306	0.8521	1.422	0.4628
		Q_4_	1.931	0.1169	1.536	2.428	<0.0001
CRP†	B	Q_2_	0.8755	0.1589	0.6413	1.195	0.4030
		Q_3_	1.370	0.1440	1.033	1.817	0.02788
		Q_4_	1.741	0.1406	1.322	2.294	<0.0001
		Q_5_	2.528	0.2414	1.575	4.058	0.00012
SII†	B	Q_2_	1.135	0.1283	0.8827	1.459	0.3227
		Q_3_	0.9654	0.1312	0.7465	1.248	0.7888
		Q_4_	1.411	0.1194	1.117	1.784	0.003938
Sex	B	Male	0.9721	0.08699	0.8197	1.153	0.7458
Smoking	B	Yes	2.755	0.1356	2.113	3.595	<0.0001
Age	B		1.034	0.006844	1.020	1.048	<0.0001
BMI	B		1.012	0.008694	0.9952	1.030	0.1617
Smoking Index	B		1.014	0.001360	1.011	1.016	<0.0001
FEV_1_/FVC	B		0.004652	0.3673	0.002264	0.009555	<0.0001

BMI – body mass index, CI – confidence interval, CRP – c-reactive protein, FEV1 – forced expiratory volume in one second, FVC – forced vital capacity, HR – hazard ratio, Q – quartile, RF – respiratory failure, SD – standard error, SII – systemic inflammation index

*Model A stands for the crude Cox proportional hazards regression model, including only the observed variables and the outcome variable. Model B stands for the adjusted Cox proportional hazards regression model, including the observed variables, common clinical covariates, and the outcome variable.

†Q_1_ as a reference for both CRP and SII.

After adjusting for clinical variables, including gender, age, BMI, smoking status, smoking index, and FEV_1_/FVC, in a multivariate Cox regression model, and adding SII as a covariate, elevated CRP levels were independently associated with increased risk of RF. C-reactive protein-Q_5_ remained a strong predictor (HR = 2.53; 95% CI = 1.57–4.06, *P* = 0.00012), and Q_4_ (HR = 1.74; 95% CI = 1.32–2.29, *P* < 0.0001) and Q_3_ (HR = 1.37; 95% CI = 1.04–1.82, *P* = 0.02876) remain significant. For SII, we only observed a statistically significant difference in the Q_4_ subgroup (HR = 1.411; 95% CI = 1.12–1.78, *P* = 0.0039) (**Figure 3**, Panel B, **Table 3**, Panel B). The PH test and Schoenfeld’s residual error plot for the main cohort showed a relatively mild time-related change in CRP subgroups (*P* = 0.0019). Global PH was acceptable (GLOBAL *P* = 0.142) (Figure S4, Panel A and Table S3, Panel A in the **Online Supplementary Document**), indicating no clear systematic trend over time in CRP quintiles for most of the follow-up period.

#### Sensitivity analysis

In the sensitivity analysis cohort, we repeated the Kaplan-Meier survival and Cox proportional hazards regression analyses comparing CRP and RF. The Kaplan-Meier curve also indicated a time-increasing tendency (Figure S2 in the **Online Supplementary Document**), and the Cox regression analysis still showed a dose-response relationship between CRP and RF (Figure S3 in the **Online Supplementary Document**). However, the Kaplan-Meier curve showed multiple crossovers during the follow-up period, and multivariate Cox analysis showed only a significant difference between the CRP-Q_3_ and -Q_4_ subgroups and the -Q_1_ group, and between the -Q_4_ group and the -Q_2_ group. However, no significant effect in the CRP-Q_5_ subgroup after adjustment for SII and the aforementioned clinical variables. The PH test showed apparent deviations at very early and late times, which likely reflect event clustering near the baseline and sparse data at the tail of the long-term follow-up, rather than meaningful violations of the proportional hazard assumption (Figure S4, Panel B in the **Online Supplementary Document**).

#### Fine-Grey model and restricted cubic spline (RCS) analysis

A Fine-Grey model was conducted to adjust for competing risks of death, and the results showed that CRP was a robust predictor of RF risk (Table S4 in the **Online Supplementary Document**). Restricted cubic spline analysis indicated a similar dose-response relationship when treating CRP as a continuous variable (Figure S5 in the **Online Supplementary Document**).

## DISCUSSION

In this up to 17.87-year cohort study of 38 933 participants with COPD, higher baseline serum CRP concentration was associated with a significantly higher risk of RF after adjustment for other covariates. The risk increased in a dose-response manner, underscoring that CRP, as a typical marker of systemic inflammation, may be associated with subsequent RF in COPD patients.

Existing studies have primarily focused on CRP and its association with COPD prognosis [21]. However, their findings are inconsistent. We found CRP may have an independent association with a higher risk for incident RF in patients with COPD, consistent with findings reported by Moy et al. (n = 173) [22] and Man et al. (Lung Health Study, n = 4803) [15]. Conversely, de Torres et al. (BODE, n = 218) [12] found no significant difference in CRP concentrations between survivors and non-survivors. Meanwhile, Grolimund et al. (PreHOSP, n = 469) reached a similar conclusion [23].

Given the contradictions among studies in this field, we hypothesise that variations in disease severity among participants may also contribute to these discrepancies. Man et al.'s study included patients with mild-to-moderate COPD [15], whereas Moy et al.'s study encompassed patients across GOLD stages I through IV, reflecting broader coverage like our study [22]. In contrast, de Torres et al. concentrated on patients with moderate to very severe disease [12], and Grolimund et al. focused exclusively on patients experiencing acute exacerbations [23]. We hypothesised that there is a relatively pronounced association between CRP levels and poor prognosis, particularly in mild-to-moderate patients with COPD. In advanced stages or acute exacerbations of COPD, such a relationship may be attenuated as inflammatory responses reach a plateau phase. Our primary cohort and sensitivity analyses also indicated differences in significance levels across CRP subgroups. The primary cohort uses FEV_1_/FVC as the criterion for COPD diagnosis, whereas the sensitivity analysis cohort relies on inpatient diagnosis, potentially indicating a more complex form of the disease; these variations in disease severity may consequently lead to differing outcomes.

In contrast to Man et al. and Moy et al., we adopted the stratification strategy proposed by Burger et al. [24]. Specifically, patients with CRP greater than 20 mg/L were classified as an independent ‘hyper-inflammatory’ subgroup in our study. We chose this design for two primary purposes. First, serum CRP in our cohort exhibited a markedly right-skewed distribution; isolating extreme values from the upper quartile can reduce misclassification bias. Second, according to existing literature and routine clinical practice, a CRP level over 20 mg/L is considered evidence of acute or heightened systemic inflammation [25,26]. In our study, the CRP-Q_5_ subgroup did not show a stable, statistically significant association with incident RF, whereas the -Q_3,4_ groups showed a more consistent association after changing the COPD definition method.

The above contradiction underscores persistent heterogeneity across COPD cohorts. As an acute-phase protein, CRP is produced by hepatocytes in response to interleukin-6 (IL-6) and other pro-inflammatory cytokines [27]. Current research has revealed two structural isoforms: pentameric CRP (pCRP) and monomeric CRP (mCRP) [28]. Pentameric CRP, the native circulating form measured in routine assays, may rise rapidly after an acute inflammatory stimulus and has a relatively stable half-life. Under oxidative stress or on damaged cell membranes, pCRP recognises lysophosphatidylcholine and converts irreversibly to mCRP, thereby exposing neo-epitopes. mCRP binds FcγIIIa/b on leukocytes, activates phagocytosis, and amplifies inflammatory response [29,30]. Approximately 6–12 hours after the ignition of acute inflammation, a second wave of pCRP binds FcγIIa and confers anti-inflammatory effects [31]. Prior biological studies have provided evidence of the biological plausibility that CRP-related inflammatory pathways may be relevant to COPD pathophysiology [32–35].

Therefore, CRP has substantial clinical value for patient management. First, laboratory examination of CRP concentration is inexpensive, widely available, and rapidly reported, making it an ideal clinical biomarker. In our research, a clear dose-response relationship between CRP and incident RF was observed, suggesting that routine CRP testing could aid physicians in strengthening the management, including oxygen, antibiotics, and inhalers, particularly in acute exacerbations. Second, CRP may guide antimicrobial stewardship, and previous work by Butler et al. [36] supports CRP-guided antibiotic prescribing. Sewell et al. [37] further demonstrated a 20% reduction in antibiotic use when CRP guidance was adopted, lowering costs and improving patient adherence.

After thoroughly discussing the clinical utility, it is equally important to conduct an objective evaluation of the study's reliability. Our key strengths include the use of the UK Biobank with up to 17.87 years of follow-up, reliable ICD-10 coding, and a broad CRP range with characteristic group division. Meanwhile, we adjusted for all-cause mortality, COPD-induced mortality, and the influence of clinical variables, as well as other inflammatory indices, when measuring CRP and RF. These features enhance the internal validity of the observed CRP-RF association. Moreover, we also conducted a sensitivity analysis using different COPD definition methods to check the CRP-RF relationship thoroughly, and a Fine-Gray analysis was carried out to reduce the complete risk of death events.

However, our research still has several limitations. First, the CRP-Q_5_ subgroup is small, which increases the standard error and may affect the interpretation of our results. Second, we did not distinguish between pentameric and monomeric CRP, and large-scale assays that measure both isoforms are not yet available [29]. Third, causality between CRP and RF cannot be inferred based on this study. Residual confounding from unmeasured mediators, such as IL-6 or fibrinogen, may persist despite adjustment for SII and standard clinical variables. Furthermore, we realised that CRP is a nonspecific biomarker whose levels can vary with COPD severity. Serial-based analyses are therefore warranted, although prior work, as well as the PH test and Schoenfeld’s residual error in our study, indicate that intra-individual CRP levels remain relatively stable over time [38,39]; therefore, estimates should be interpreted cautiously, especially in the high-concentration subgroup of CRP. Moreover, we still have limitations in the thorough screening and assessment of baseline information, including comorbidities within and outside the respiratory system, clinical status, and incident RF scenarios. The methods used to define COPD and RF based on ICD-10 codes may lead to misclassification. Data from the UK Biobank also introduces healthy-participant bias, which may limit the extrapolation of our study to intensive care patients. Moreover, the possibility of death competition might reduce the cumulative incidence of RF remains, even if we had attempted to account for mortality. In general, such biases may have a subtle impact, but are unlikely to reverse the direction of the CRP-RF relationship.

Owing to its low cost and accessibility, CRP may potentially play a crucial role as an RF biomarker in the management of COPD. Isoform-specific longitudinal profiling within a single COPD cohort could map distinct inflammatory trajectories and relate them to RF and all-cause or COPD-induced mortality. A full appreciation of the diagnostic and therapeutic potential of CRP, particularly mCRP, may advance personalised management of COPD worldwide, in our view.

## CONCLUSIONS

C-reactive protein concentration may be independently associated with incident high-risk RF in patients with COPD. Future trials may reveal the exact cut-off point, indicating a high possibility of RF.

## Additional material


Online Supplementary Document
