# Supplementary material for: C-reactive protein predicts respiratory failure in chronic obstructive pulmonary disease: a cohort analysis from the UK Biobank
Source: J Glob Health. 2026 Feb 27;16:04061. doi: 10.7189/jogh.16.04061 (PMC12947716; doi:10.7189/jogh.16.04061)
Supplement: Online Supplementary Document [file jogh-16-04061-s001.pdf]

Supplemenet to: Zhang BY, Dai ZS, Jiang Q. Zhao R. Chen Y. C-reactive protein predicts respiratory failure in chronic obstructive pulmonary disease: a cohort analysis from the UK Biobank. J Glob Health. 2026;16:04061.

Table of contents (*use Ctrl + click to navigate*)

Supplemental Methods **p.2**

Supplemental Figures **p.3**

Supplementary Online Figure 1. Cohort Flowchart p.3

Supplementary Online Figure 2. Kaplan-Meier Curve and Pairwise Analysis After Bonferroni Correction of Sensitivity Analysis Cohort p.4

Supplementary Online Figure 3. Cox Regression Analysis of Sensitivity Analysis Cohort p.5

Supplementary Online Figure 4. Schoenfeld's Residual Error Plot p.6

Supplementary Online Figure 5. Restricted Cubic Spline Curve Between CRP and RF p.7

Supplemental Tables **p.8**

Supplementary Online Table 1. Adherence to *JoGH's Guidelines for Reporting Analyses of Big Data Repositories Open to Public* (GRABDROP) p.8

Supplementary Online Table 2. Detailed Judgment Rules of Initial Smoking Status "Unknown" p.10

Supplementary Online Table 3. The PH Test for Main Cohort and Sensitivity Analysis Cohort p.11

Supplementary Online Table 4. Fine-Gray Test on the Main Cohort p.12

Supplemental Methods

Data collection

Blood samples from participants were added to serum separation tubes and stored upside down at the assessment center, thoroughly mixed with preservative and/or anticoagulant agents. Subsequently, the serum tubes were left to coagulate at room temperature for 30 min. Samples were sent to the UK Biobank central processing and filing laboratory, which took an average of 24 ± 2.5 hours. Highly automated and customized industrial immunoturbidimetry equipment (up to the ISO 17025:2005 international standard) was used in the relevant facilities, and a standardized sampling method was employed to minimize epidemiological bias attributable to geographical location, collection date, and other factors.

Ten immunoanalyzers (6x DiaSorin Liaison XL & 4x Beckman Coulter DXI 800 and 4 clinical chemistry analysers (2x Beckman Coulter) were used AU5800 & 2x Siemens Advia 1800)) was utilized to analysis CRP concentration, and the lower limit of detection for CRP was 0.08mg/L, and the upper limit of detection was 80mg/L; The detection limit of ALB is 15g/L, and the detection limit is 60g/L. The precision, accuracy, bias, linearity, and reportable range of the relevant instruments have been tested.

Blood drawn from each subject was deposited in 4mL EDTA sampling tubes, and standard hematological tests were performed on fresh whole blood within 24 h of blood collection. Four Beckman Coulter LH750 quantitative automatic hematology analyzers and leukocyte differential counters located in the UK Biobank central laboratory were used to carry out relevant tests.

Judgement criteria of the history of smoking

In order to improve the accuracy of judging smoking history and take into account the factors of population size, we used “Current tobacco smoking”, “Past tobacco smoking”, “Ever smoked”, “Smoking status”, and other variables to preliminarily judge smoking history as “Yes” or “No”. Smoking history was classified as “Unknown” when there was a conflict among the above variables, and more detailed judgment criteria are provided in Supplementary Online Table 2, based on the actual conditions of our data.

Moreover, we applied a correction using “Light smokers, at least 100 smokes in lifetime” for those who had been classified as “Yes” or “No” in the preliminary judgment to improve decision accuracy.

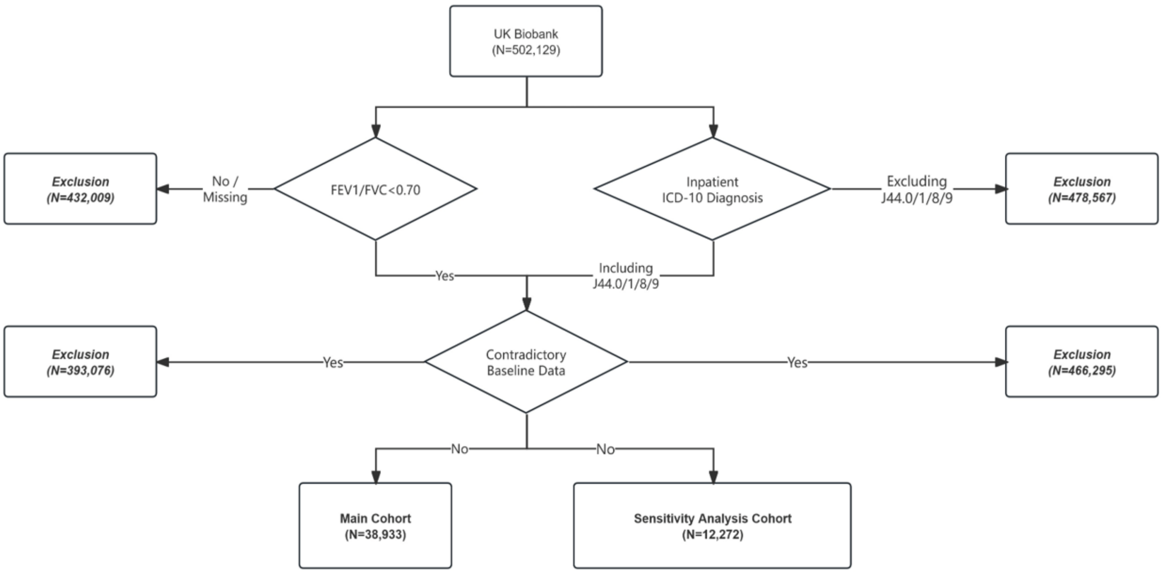

Supplementary Online Figure 1. Cohort Flowchart

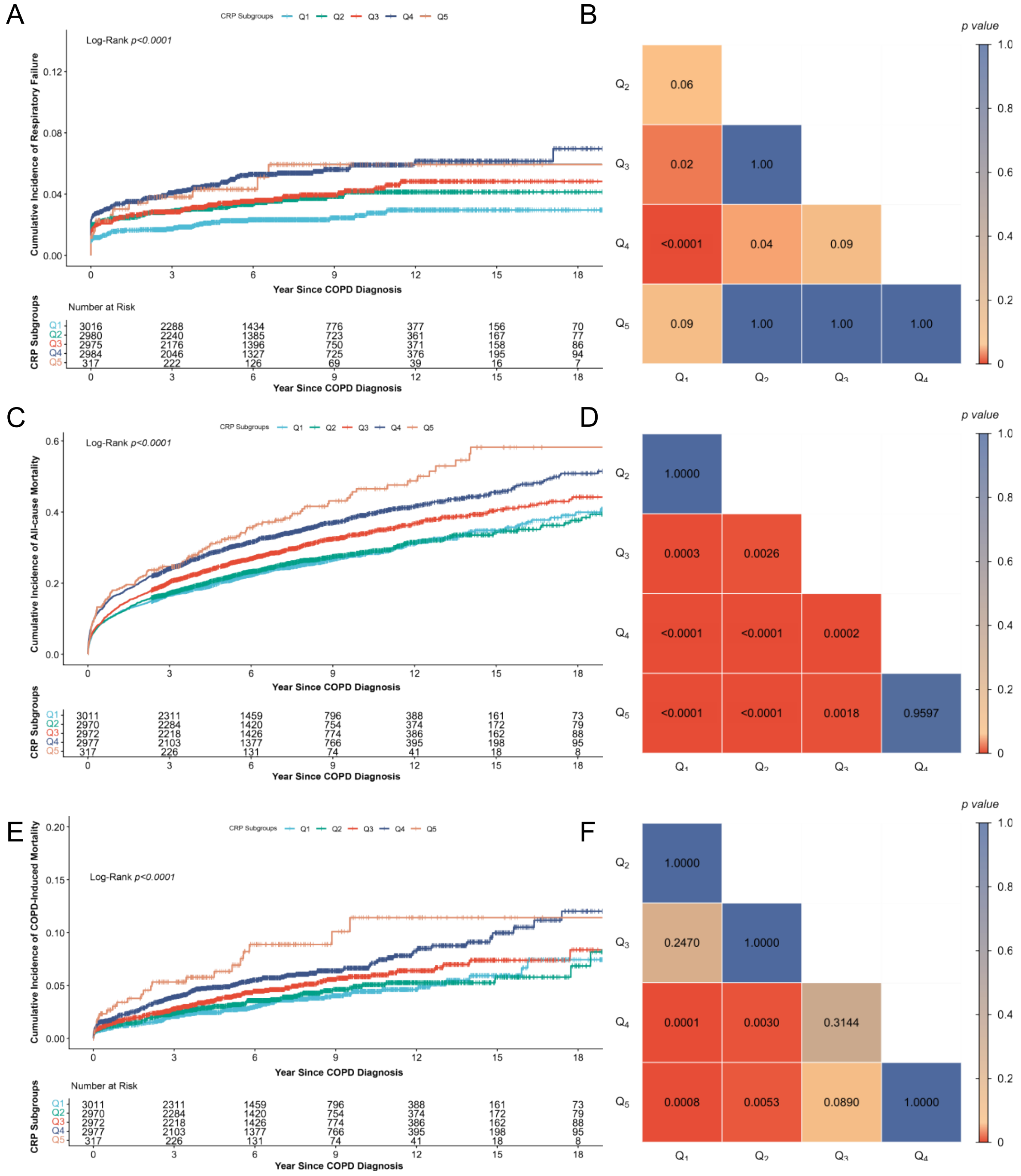

Supplementary Online Figure 2. Kaplan-Meier Curve and Pairwise Analysis After Bonferroni Correction of Sensitivity Analysis Cohort

Fig. S2A. Kaplan-Meier curve of CRP on RF, **Fig. S2B.** Pairwise comparison after Bonferroni correction of CRP on RF, **Fig. S2C.** Kaplan-Meier curve of CRP on all-cause mortality, **Fig. S2D.** Pairwise comparison after Bonferroni correction of CRP on all-cause mortality, **Fig. S2E.** Kaplan-Meier curve of CRP on COPD-induced mortality, **Fig. S2F.** Pairwise comparison after Bonferroni correction of CRP on mortality.

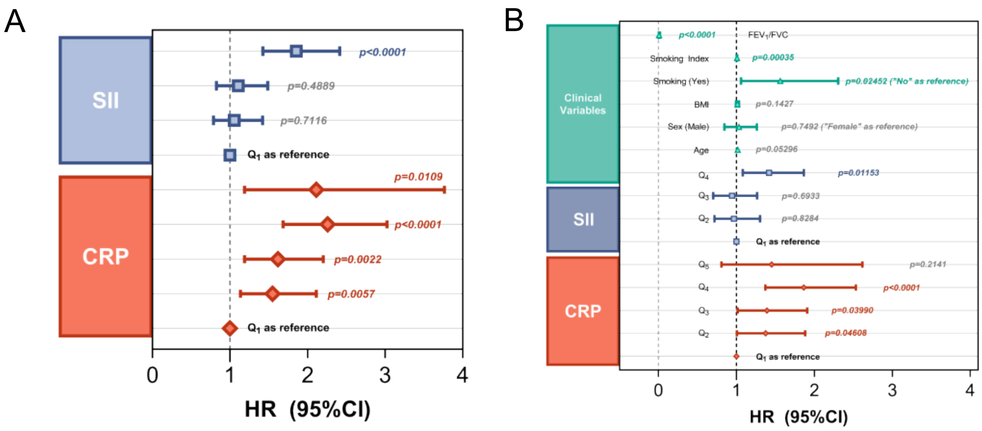

Supplementary Online Figure 3. Cox Regression Analysis of Sensitivity Analysis Cohort

Fig. S3A. Univariate Cox regression analysis of CRP and SII, **Fig. S3B.** Multivariate Cox regression analysis of CRP adding SII as covariate after correction with clinical variables.

A

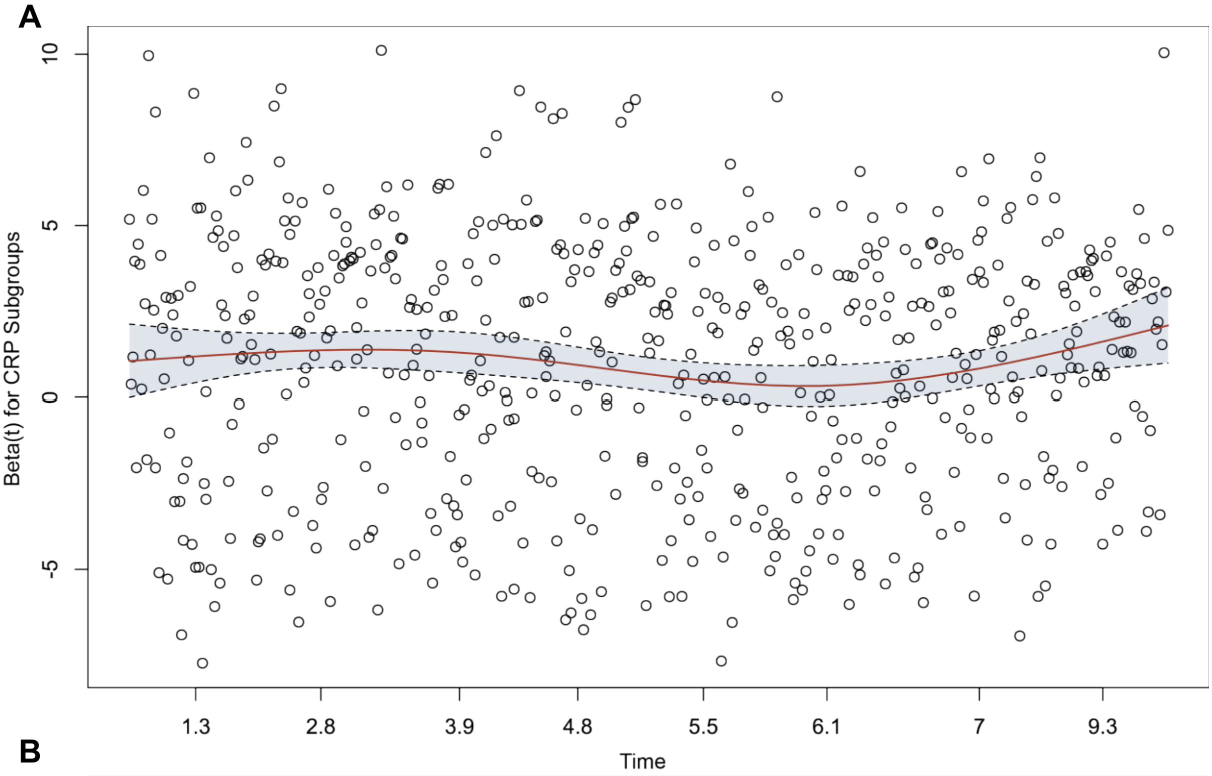

B

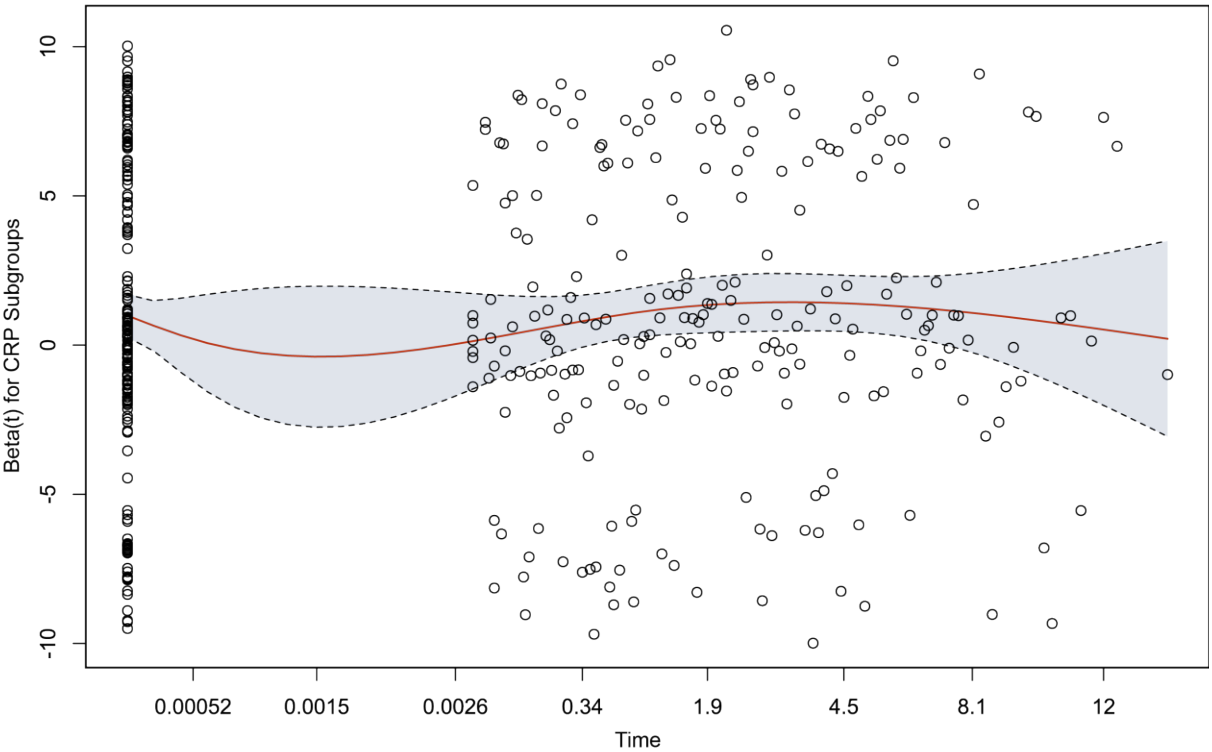

Supplementary Online Figure 4. Schoenfeld's Residual Error Plot

Fig. S4A. Schoenfeld's residual error plot for the main cohort, **Fig. S4B.** Schoenfeld's residual error plot for the sensitivity analysis cohort.

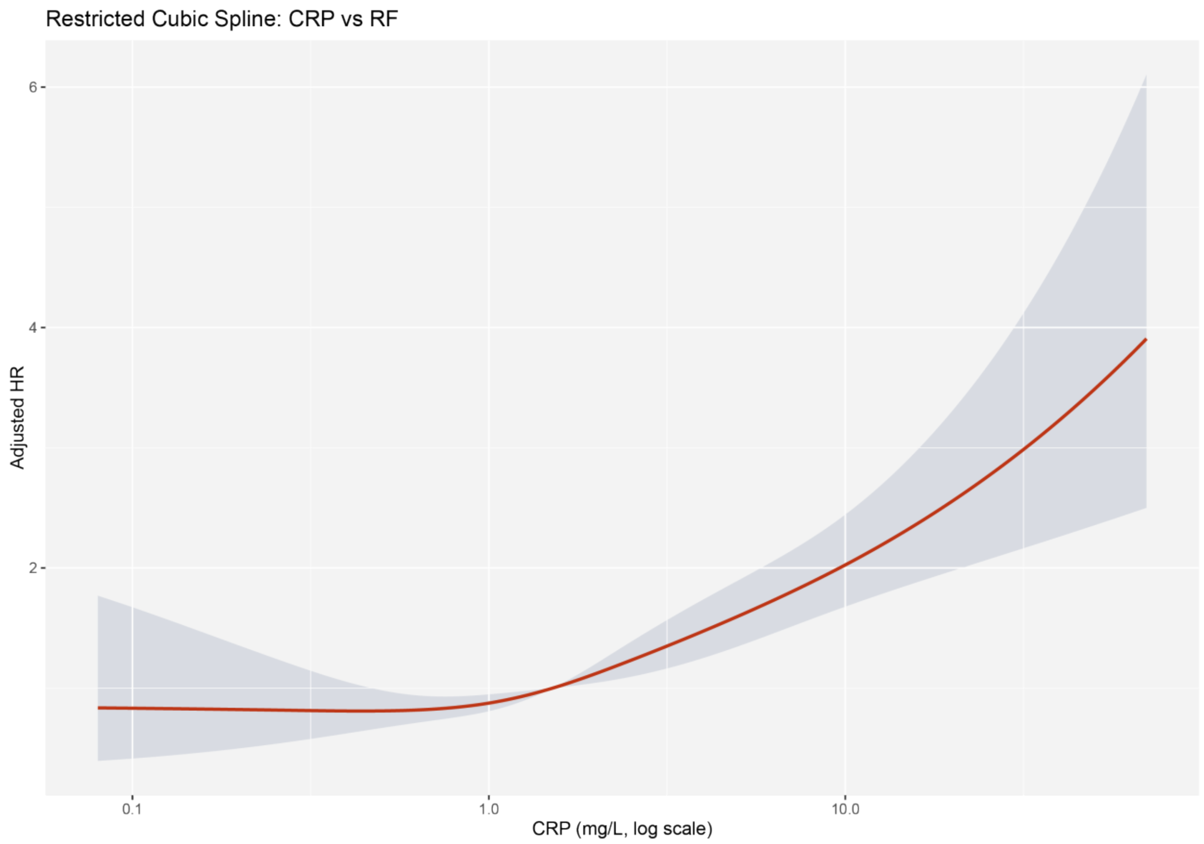

Supplementary Online Figure 5. Restricted Cubic Spline Curve Between CRP and RF

Supplementary Online Table 1. Outline of JoGH guideline items

JoGH guideline item	Author's Response
1. Please list all papers published by each co-author in previous 3 years that were based on secondary analysis of a big data repository	Boyan Zhang. N/A<br>Zhongshang Dai. N/A<br>Qi Jiang. N/A<br>Rui Zhao. N/A<br>Yan Chen. N/A
2. Please explain the key elements of your study design and the use of the available datasets that make your study an original scientific contribution	Our study is a large-scale (N = 38,933), long-term follow-up cohort study. Leveraging the scale of the UK Biobank dataset, we can observe the long-term impact of baseline CRP on adverse outcomes with high statistical power, which is often limited in smaller clinical cohorts. Unlike prior studies that focused on all-cause mortality or acute exacerbations, we defined incident respiratory failure as the primary outcome, which offers new insights into the link between systemic inflammation and respiratory decompensation. Moreover, we divided patients with CRP > 20 mg/L into an individual group based on large-scale participation in the UK Biobank. This allowed us to characterize a "hyper-inflammatory" phenotype and identify a dose-response relationship that conventional quartile-based analyses might obscure. Finally, we constructed a robust multivariate Cox regression model, adjusted not only for other inflammatory markers but also for comprehensive clinical confounders (e.g., age, smoking status, BMI, lung function, etc.), addressing potential residual confounding common in previous observational studies.
3. Please list all publications that addressed similar research questions in the same dataset and indicate where you cited them in your paper	We conducted a comprehensive search of medical databases, including PubMed and Web of Science. We confirmed that a limited prior study has specifically investigated the association between baseline CRP thresholds and incident Respiratory Failure in COPD patients within the UK Biobank. However, several similar studies used the same dataset to address related topics, which we cite to contextualize our findings.<br><br>1. He J, Li M, Luo P, et al. The joint effect of triglyceride-glucose index and C-reactive protein levels on the risk of chronic obstructive pulmonary disease. a prospective cohort study. *Lipids Health Dis*. 2025;24(1).309. Published 2025 Oct 6. doi.10.1186/s12944-025-02732-1<br>This research focused on the individual and joint effects of Triglyceride-glucose index (TyG) and CRP upon COPD risk, proving that the simultaneous elevation of both TyG and CRP significantly increased the risk of COPD in the healthy population. The distinction between our research and this paper concerns the population we selected. He et al. focused on the joint effect of CRP and TyG in the general population. In contrast, our study investigates the prognostic value of CRP specifically for incident respiratory failure within an established COPD cohort.<br><br>What's more, this article was published on October 6, 2025, which was after the submission of our original manuscript. Therefore, it was not included in the previous version of the paper. However, we have now cited this recent study in the Introduction section (Reference 14) of the revised manuscript.<br><br>2. Ji M, Du L, Ma Z, et al. Circulating C-reactive protein increases lung cancer risk. Results from a prospective

	cohort of UK Biobank. *Int J Cancer*. 2022;150(1).47-55. doi.10.1002/ijc.33780.<br>This research mainly focused on the relationship between CRP and lung cancer, which is also a common comorbidity in COPD patients. However, our research primarily focused on patients with COPD at the primary stage.
4. Please explain how you addressed multiple testing through an appropriately rigorous statistical threshold and indicate this in the methods section	We have conducted rigorous statistical analysis. In the Kaplan-Meier survival analysis, when comparing survival differences between specific CRP subgroups, we applied the Bonferroni correction to the Log-Rank tests to rigorously control the family-wise error rate. This is explicitly stated in the Methods section *(Subsection 3.4.3)*. Regarding the primary multivariate Cox regression analysis, as this was a hypothesis-driven investigation based on a specific a priori assumption, we adhered to standard epidemiological practice by reporting 95% confidence intervals alongside Hazard Ratios. We focused on the magnitude and precision of effect estimates rather than applying p-value penalties, thereby minimizing the risk of Type II errors in detecting clinically relevant associations.
5. Please declare to what extent have AI chatbots been used in developing your paper and to which parts of the paper did they contribute	Artificial Intelligence chatbots were used exclusively to refine language, improving readability and grammatical accuracy. We declare that AI tools were **NOT** used for data analysis, statistical processing, image generation, or the formulation of scientific hypotheses and conclusions. The authors have reviewed the final manuscript meticulously and take full responsibility for the content and integrity of the work.

Supplementary Online Table2. Detailed Judgment Rules of Initial Smoking Status "Unknown"

Current tobacco smoking	Past tobacco smoking	Ever smoked	Smoking status	Light smokers, at least 100 smokes in lifetime	Pack years of smoking	Smoking
Unknown / Prefer not to answer					Detailed values	Yes
No	I have never smoked	No	Current / Previous	-	N/A	*Censored*
No	Unknown / Prefer not to answer				N/A	No
Unknown / Prefer not to answer	I have never smoked	Unknown / Prefer not to answer			N/A	No
Unknown / Prefer not to answer			Never	Unknown / Prefer not to answer	N/A	No

Supplementary Online Table 3a. The PH Test for the Main Cohort

Variable	χ^2	df	*p-value*
CRP subgroups	11.7381	4	*0.019*
SII subgroups	2.0798	3	*0.556*
Age	0.0424	1	*0.837*
Sex	0.0298	1	*0.863*
BMI	0.3381	1	*0.561*
Smoking status	3.1960	1	*0.074*
Smoking Index	2.1004	1	*0.147*
FEV_1/FVC	0.5231	1	*0.470*
**GLOBAL**	18.4219	13	*0.142*

Supplementary Online Table 3b. The PH Test for the Sensitivity Analysis Cohort

Variable	χ^2	df	*p-value*
CRP subgroups	3.626	4	*0.4590*
SII subgroups	4.141	3	*0.2466*
Age	0.478	1	*0.4893*
Sex	1.209	1	*0.2716*
BMI	7.480	1	*0.0062*
Smoking status	2.032	1	*0.1540*
Smoking Index	1.489	1	*0.2223*
FEV_1/FVC	10.823	1	*0.0010*
**GLOBAL**	28.522	13	*0.0076*

Supplementary Online Table 4. Fine-Gray Test on the Main Cohort

Variable	SHR	CI low	CI high	*p-value*
CRP-Q_2	0.8856	0.6481	1.211	*0.4473*
CRP-Q_3	1.398	1.056	1.850	*0.01924*
CRP-Q_4	1.798	1.362	2.372	*<0.0001*
CRP-Q_5	2.654	1.658	4.249	*<0.0001*
Age	1.032	1.018	1.046	*<0.0001*
Sex	0.9561	0.8015	1.140	*0.6179*
BMI	1.012	0.9925	1.031	*0.2322*
Smoking Status	2.726	2.097	3.543	*<0.0001*
Smoking Index	1.013	1.011	1.016	*<0.0001*
FEV_1//FVC	0.004816	0.002541	0.009129	*<0.0001*
